# Successful Pregnancy After Combined Liver and Renal Transplantation in a Patient With Acute Intermittent Porphyria

**DOI:** 10.1155/crit/5522456

**Published:** 2025-06-19

**Authors:** Petro E. Petrides, Joachim Andrassy, Markus Guba, Manfred Stangl, Olivia Bronisch, Maria K. Beykirch

**Affiliations:** ^1^IPNET Center Munich, Hematology Oncology Center, Munich, Germany; ^2^LMU University of Munich Medical School, Munich, Germany; ^3^Transplantation Unit, LMU University of Munich Medical School, Klinikum Grosshadern, Munich, Germany

## Abstract

Acute intermittent porphyria is a rare inborn disease of porphyrin metabolism which can cause severe abdominal pain attacks and neurological symptoms. Here, we report a patient with a 20-year history of severe chronic manifestations of acute intermittent porphyria that led to end-stage renal disease and liver function impairment. Since only transplants can cure both disease manifestations, a combined liver and renal transplantation was performed. The patient recovered so well that she delivered a very low birth extreme premature baby 16 months later which developed normally over the next 5 years. Our case represents the third case in the literature with a successful combined liver/renal transplantation of a patient with acute porphyria. Thus, transplantation seems to be a viable backup option, should novel therapies such as siRNA treatment with givosiran fail.

## 1. Introduction

Acute porphyrias are rare inherited disorders of heme metabolism characterized by acute abdominal pain and neurological complications [1]. Symptomatic patients may require heme infusions. A small minority of patients experience recurrent attacks, which historically required long-term heme therapy for many years. These attacks can be so debilitating that chronic use of morphine becomes necessary. The only definitive cure at present is a liver transplant. Long-term complications include renal disease [2], liver fibrosis [3], and hepatocellular carcinoma [4]. However, newer therapies, such as givosiran, may help prevent these complications [5].

## 2. Case History

We report the case of a patient from Afghanistan who had emigrated to Germany. At the age of 16, in December 1996, she suddenly developed abdominal and back pain. In March 1997, she was diagnosed with acute intermittent porphyria (AIP) with a porphobilinogen (PBG)-deaminase activity of 48% and the c.445C>T, p.Arg199X mutation. Since her diagnosis, she has required regular heme infusions every 3 months as part of our Munich cohort of patients with acute porphyria [6].

Over the following years, the frequency and severity of her attacks, characterized by back, abdominal, and stomach pain, progressively worsened, necessitating monthly heme infusions. Between these attacks, she mainly experienced fatigue. Any attempt to extend the interval between infusions aggravated her symptoms. In 2001, she became pregnant but requested an abortion in December that year. In December 2002, after a second pregnancy, she gave birth to a healthy son. In August 2003, hormonal ablation using the gonadorelin analogue goserelin (3.6 mg/month) to prevent further porphyria attacks was attempted but proved unsuccessful [7].

A major issue contributing to her acute attacks was her low body weight (40 kg, height 162 cm, BMI 15.2 kg/m^2^), so parenteral nutrition was often necessary. In February 2011, she was offered liver transplantation as a curative option, but she declined the offer.

**Figure 1:**
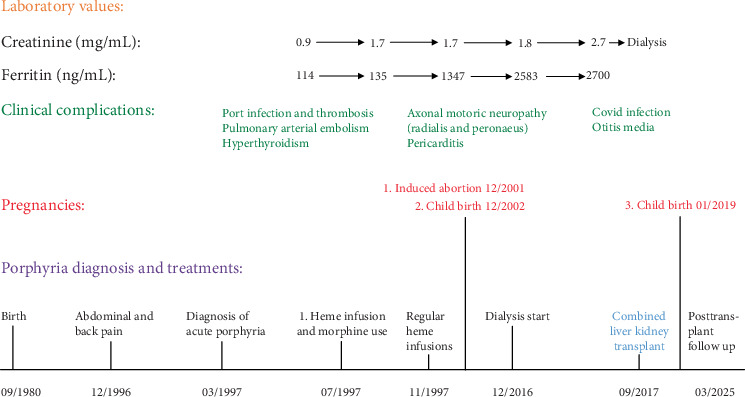
Clinical course of our AIP patient from onset of symptoms until present. Note: year axis not on scale.

Chronic administration of iron-containing heme resulted in elevated ferritin levels (Figure 1), prompting the initiation of iron mobilization therapy with the oral iron chelator deferasirox. However, this treatment had to be discontinued because of side effects such as diarrhea and increasing creatinine levels. Phlebotomy was not considered because the patient's hemoglobin level was just above 10 g/dL. Consequently, her ferritin levels continued to increase, reaching 2700 ng/mL by July 2015. Owing to her frequent need for pain management, she was regularly seen in the pain clinic and was prescribed tramadol, with subcutaneous morphine (5–10 mg) administered during acute attacks. Over time, she developed opioid tolerance, complicating effective pain relief. Simultaneously, her creatinine levels increased to 1.8 mg/dL (glomerular filtration rate (GFR) 36 mL/min), further rising to 2.7 mg/dL (GFR 22 mL/min) by July 2015 (Figure 1). Eventually, she developed end-stage renal disease (ESRD) by December 2016.

## 3. Treatment

With informed consent and a multidisciplinary approach, a combined liver and kidney transplantation was carried out in September 2017. The operation took 7.5 h: first, the liver was transplanted and then the kidney. The HLA type of the donor was A9, A9, A24, B7, B8, Cw7, Cw7, DR3, DR8, DR52, DQ2, and DQ4; the one of the recipient was A∗ 03,30; B∗ 13,51; C∗ 06,16; DRB1∗ 04,11; and DQB1∗ 03,03, indicating a complete mismatch. Immunosuppression with tacrolimus, azathioprine, and steroids was started.

Histological analysis of the explanted liver showed severe hemosiderosis, although no fibrosis was present at that time.

## 4. Outcome and Follow-Up

Since the liver transplantation, our patient has been free of acute attacks. Her urine PBG and 5-aminolaevulinic acid values have returned to normal. However, PBG-deaminase in erythrocytes remains reduced at 48% owing to the persistent genetic defect in the bone marrow.

Less than a year after the transplant, the patient became pregnant again while on immunosuppressive therapy with 6.5 mg of tacrolimus daily. At gestational week 25 + 0, she was diagnosed with early preeclampsia. The baby was delivered by secondary caesarean section at gestational week 29 + 4 in January 2019, with a low birth weight of 1090 g and placental infarction. To prevent respiratory distress syndrome in the newborn, she received steroid prophylaxis.

**Figure 2:**
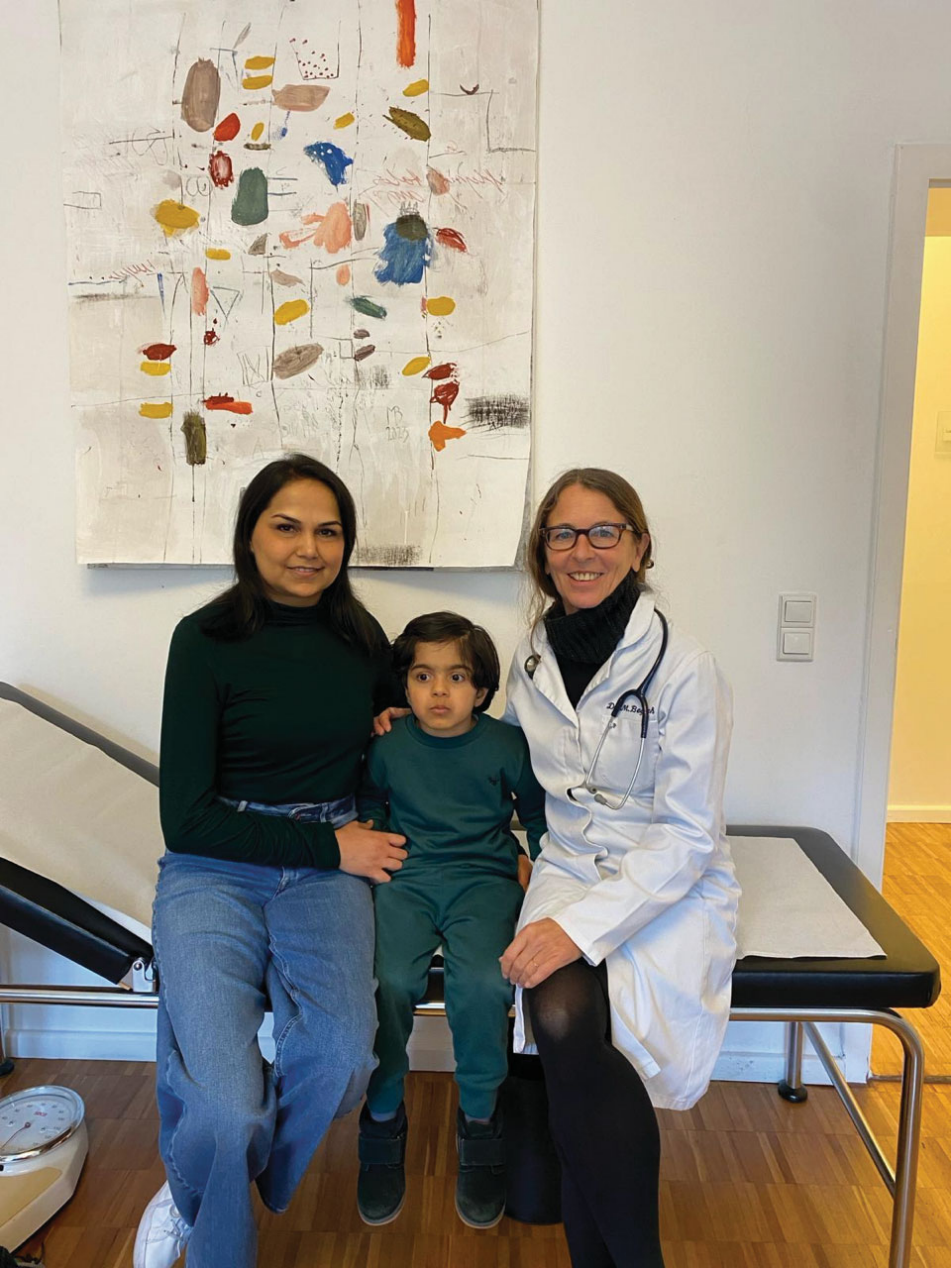
The patient with her 5-year-old boy and her physician Dr. Beykirch.

Regular follow-up over the past 8 years has shown that the patient maintains a good performance status, with a body weight of 49 kg and a stable creatinine level of 1.5 mg/dL (GFR 40 mL/min, normal above 60 mL/min). However, her hemoglobin level was temporarily low at 7.6 g/dL (normal range 12–16 g/dL) because of hypochromic anemia, which was treated with parenteral iron infusions (ferritin 7 ng/mL, normal > 50 ng/mL). Her child, who was 6 years old at the time of this writing, has developed normally (Figure 2).

During the last follow-ups in 01/2025 and 03/2025, her hemoglobin values were between 11.9 and 13.1 g/dL and her creatinine levels between 1.3 and 1.5 mg/dL (GFR 42–50 mL/min) with a discrete albuminuria of 40 mg/g creatinine. She was negative for anti-HLA antibodies as well as negative on BK virus and CMV-PCRs.

Taking 50-mg azathioprine, 5-mg prednisone, and 2.5-mg tacrolimus per day, her tacrolimus levels (LC-MS/MS) were between 3.2 and 4.9 ng/mL. In addition, her antihypertensive treatment consisted of bisoprolol (5 mg), lercanidipine (10 mg), and candesartan (8 mg).

She reported that her well-being is mainly dependent upon her iron supplies.

## 5. Discussion

Most patients with acute porphyrias, such as AIP, experience only one major attack during their lifetime. However, a small minority of patients, usually females aged 20–40 years, suffer from chronic attacks that can last for years. These patients often require repeated iron-containing heme infusions and strong pain medications. In some patients, the chronic nature of their illness can lead to liver and/or renal function impairment. Liver damage may be caused by chronic inflammation and fibrosis due to iron accumulation because of heme infusions [8] or the disease itself [9, 10]. Chronic renal damage occurs in 50% of the patients with symptomatic AIP, many of whom also have hypertension [2]. Owing to the invasive nature of liver transplantation, the need for lifelong immunosuppression, and the shortage of donors, it is considered a last resort option for patients who continue to experience frequent attacks and no longer respond to pharmacological therapies. Moreover, transplant-associated complications such as hepatic artery thrombosis can occur. The first liver transplantation in a patient with AIP was reported 20 years ago [11], and since then, this treatment has been performed several times [12].

A large European review reported data from 38 patients with AIP who underwent liver transplantation: 89% of the patients were female, with a median age of 37 years [13]. All patients experienced relief from acute attacks, and their survival rates were comparable to those of patients who had undergone transplantations for other similar diseases. While neurological symptoms improved after liver transplantation, renal function continued to decline in some cases.

Renal transplants are performed less frequently than liver transplants in patients with AIP, typically after appropriate pretransplant evaluation [14]. The outcomes of 11 patients with AIP and ESRD with a median age of 35 years were documented in the French Porphyria Center Registry [15]. In this cohort, two patients died from different causes, and no episodes of rejection or significant adverse events were observed. The authors concluded that renal transplantation is the treatment of choice for patients with AIP with ESRD, as posttransplantation care is simple, drug management is easy, and quality of life improves significantly.

In extremely rare cases, chronic illness can lead to both liver and renal function impairments, requiring the replacement of both organs. This dual transplant approach is occasionally used in rare metabolic diseases where ESRD is caused by a genetic defect in the liver [16]. To date, there have been only two reports of such a combined procedure in patients with acute porphyrias [17, 18].

After transplantation, pregnancy carries an increased risk [19]. However, in patients with AIP, successful pregnancies can occur not only after liver [20] or renal transplantation [21] but also, as observed in our patient, after a combined renal and liver transplant.

Recently, novel therapies such as siRNA technology with givosiran have become available for treating patients with recurrent attacks [5]. These therapies hold promise in protecting patients from the risks of organ involvement, potentially reducing the need for transplant procedures in the future.

## Data Availability

The data that support the findings of this study are available from the corresponding author upon reasonable request.

## General Statement

*Key Clinical Message.* Porphyrias are metabolic diseases which can lead to long-term organ impairment. Transplantation is the last resort for therapy. After a 20-year history, our patient received a liver–renal transplant: this procedure cured the disease and restored renal function. Quality of life was dramatically improved so that the patient became pregnant thereafter.
